# Clinical, Hematological, and Biochemical Profile in Seropositive Dengue Cases at a Tertiary Care Hospital in Nepal

**DOI:** 10.1155/jotm/7786856

**Published:** 2024-12-24

**Authors:** Eans Tara Tuladhar, Pratibha Kandel, Sujata Baidya, Smrity Rajkarnikar, Moniya Tamrakar, Gautam Rijal, Raju Kumar Dubey, Aseem Bhattarai, Mithileshwer Raut, Apeksha Niraula, Ramesh Kumar Maharjan, Vijay Kumar Sharma

**Affiliations:** ^1^Department of Clinical Biochemistry, Maharajgunj Medical Campus, Institute of Medicine, Maharajgunj, Kathmandu, Nepal; ^2^Department of Emergency Medicine, Tribhuvan University Teaching Hospital, Maharajgunj, Kathmandu, Nepal; ^3^Department of General Surgery, Princess of Wales Hospital, Bridgend, Wales, UK

**Keywords:** dengue, leucocytes, thrombocytopenia

## Abstract

**Background:** Dengue virus infection is a major source of morbidity and mortality in the majority of tropical and subtropical nations. In Nepal, the first case of dengue was reported in 2004, followed by numerous outbreaks exerting a critical impact on public health. This study aims to describe the clinical and laboratory characteristics of dengue patients visiting a tertiary care hospital to see the trend of presentation.

**Method:** Hospital based cross-sectional study was conducted among diagnosed cases of dengue from April 2023 to September 2023. A total of 692 patients undergoing testing by commercially available dengue rapid diagnostic tests were recruited and categorized dengue positive (if NS1 and/or IgM positive) and dengue negative (NS1, IgM, and IgG all negative or only IgG positive). The dengue-positive cases were further subdivided into three groups (only NS1 positive, only IgM positive, both NS1 and IgM positive). Additionally, biochemical and hematological analyses were performed, and results were compared between positive and negative cases by using Mann–Whitney U test while subgroups of dengue-positive cases were compared using Kruskal–Wallis H test.

**Results:** Most common symptoms were fever (94.5%) followed by headache (79.8%) and myalgia (74.7%). Among 346 dengue-positive subjects, 53.2% (*n* = 184) were NS1-only positive, 21.7% (*n* = 75) were IgM-only positive, and 25.1% (*n* = 87) were both NS1+IgM positive. Thrombocytopenia (*n* = 179, 51.7%), leucopenia (*n* = 99, 28.6%), increased SGPT (*n* = 182, 52.6%), increased SGOT (*n* = 188, 54.3%) were seen among dengue positive patients. Leukopenia was more severe in patients with only NS1 positive cases (*p* = 0.008) whereas thrombocytopenia (*p* ≤ 0.001) was more severe in patients with both IgM and NS1 positive cases.

**Conclusion:** Our study depicted there is a marked alteration in biochemical and hematological parameters specifically thrombocytopenia, leukopenia, increased transaminase levels, and high prothrombin time seen in dengue positive cases.

## 1. Introduction

Dengue fever is a mosquito-borne arboviral disease caused by four serotypes of dengue virus (DEN-1 to DEN-4), member of *Flaviviridae* family. The mosquitos as dengue vectors are *Aedes aegypti* and, to a lesser extent, *Aedes albopictus* [1]. About half of the world's population is at risk with dengue with an estimated 100–400 million infections occurring every year [2]. Globally, 92 nations and territories reported more than six million dengue cases and more than 6000 dengue-related fatalities in 2023 [3]. The dengue is endemic in regions of Africa, the Americas, the Eastern Mediterranean, Western Pacific region, and Southeast Asia. The dengue virus is known to be endemic in 10 of the 11 Member States of the WHO Southeast Asia region that includes Nepal and its neighboring country, India. Dengue cases have increased significantly in 2023 compared to prior years in several countries, including Bangladesh and Thailand in SEAR (South East Asian Region) while three countries from European region also reported outbreak of dengue in 2023. Furthermore, changes in dengue patterns both temporally and spatially were noted in 2022 and continued in 2023 [4]. According to the Epidemiology and Disease Control Division (EDCD) of the Ministry of Health and Population, a total of 77 districts in Nepal have reported 51,243 cases of dengue between January 1 and December 15, 2023, with 20 confirmed fatalities (CFR = 0.05%). The highest number of cases in 2023 have been reported from Sunsari district (*n* = 16,174; 31.6%) which was followed by Tanahun (*n* = 7193; 14.0%), Jhapa (*n* = 3825; 7.5%), Dhading (*n* = 3239; 6.3%), Kaski (*n* = 3069; 6.0%), Morang (2328; 4.5%), and Kathmandu district (2076; 4.1%) [5].

Dengue virus is a single-stranded RNA virus measuring 50 nm in length, the genome of the dengue virus (DENV) is around 11 kb in length [6]. Three structural genes, which code for the proteins capsid (C), membrane (M), and envelope (E), and seven nonstructural (NS) genes, which code for the proteins NS1, NS2A, NS2B, NS3, NS4A, NS4B, and NS5 are present in the virus [7]. DENV is detected by a variety of diagnostic techniques, including virus-specific serological testing, molecular detection, and viral isolation [8]. In a country with limited resources like Nepal, gold standard diagnostics such as nucleic acid amplification assays are not easily accessible in hospitals and clinics. For the diagnosis of dengue, several laboratories use immunochromatography (ICT)-techniques based rapid diagnostic kits which has fast turnaround time. In the first 0–7 days after the beginning of symptoms, NS1-Ag detection can be just as sensitive as a molecular test; but, in secondary infections, detection may be limited by IgG antibodies from an earlier infection [9]. The three indicators that are most frequently utilized in serological testing are IgG for past infections, NS1-Ag and IgM for acute infections. In contrast to IgM, which is detectable 4-5 days after symptoms begin and may continue to be produced for up to 3 months post onset, NS1-Ag may be detected from the first 0–9 days of symptom onset and IgG levels can be detected throughout the life starting from 10 to 14 days of postinfection [10]. Although active dengue detection through ICT is simple, quick to use, and has a low sensitivity and low specificity, it also has a higher cross-reactivity that increases the number of false positives which brings in the hematological and biochemical indicators supporting test for dengue diagnosis [11–14]. Moreover, there is paucity in the studies that have compared and linked laboratory data to dengue serological markers.

The blood profile of dengue patients begins to change as soon as fever appears. Due to plasma leakage, thrombocytopenia often develops in 3–8 days, followed by leukopenia and hemoconcentration [15]. Dengue is more frequently associated with acute febrile leukopenia and positive tourniquet tests than with influenza, enteroviruses, and leptospirosis [16]. Acute kidney injury (AKI) is one of the least studied complications but carrying prolonged hospital stay and high mortality in dengue cases. Renal injury in dengue fever is likely due to hemodynamic fluctuations that can occur during the clinical course of the disease due to cytokine storm [17–19]. This cytokine storm also leads to activation of the complement system and endothelial damage resulting in increased vascular permeability with consequent hemoconcentration causing shock, leading to reduced renal perfusion and kidney injury [9, 10, 15].

A precise clinical and laboratory profile is essential for an accurate diagnosis and efficient patient care. Therefore, the goal of this study is to describe the clinical and laboratory characteristics of dengue patients visiting our hospital in 2023.

We wanted to see any drift or consistency in clinical and laboratory results of dengue cases of 2023 with previous reports.

## 2. Materials and Methods

### 2.1. Patient Selection

This was a hospital based cross-sectional study conducted among 692 patients (346 dengue-positive cases and 346 dengue-negative cases) who were investigated for dengue markers in Emergency laboratory of Tribhuvan University Teaching Hospital (TUTH) during the study period of six months (April 2023 to September 2023) after taking informed consent from the participants by convenient sampling.

The hematological, liver function test and renal function test results were obtained from the laboratory databank. The clinical profile data were collected from the patient record file. The recruited patients were categorized into two groups: dengue positive (if NS1 and/or IgM positive) and dengue negative (NS1, IgM, and IgG all negative or only IgG positive). The dengue-positive cases were further subdivided into three groups (only NS1 positive, only IgM positive, both NS1, and IgM positive). Patients with fever with other known infectious etiology, patients of known chronic liver disease, and chronic kidney disease were excluded from the study.

### 2.2. Sample Size (SS) Calculation

As the study population is infinite, the SS was calculated using the following formula:

(1) $$\begin{array}{c} \text{SS}=\frac{z^{2}\left(p\right)\left(q\right)}{d^{2}}, \end{array}$$

SS = sample size

*q* = 1 − p

*z* = standard normal prevalence at 95% Confidence Interval = 1.96.

Based on the similar study done in Nepal by Saud et al., the prevalence (*p*) of dengue was 65.7% [20].

*d* = Type I error = 5% = 0.05

(2) $$\begin{array}{c} \text{SS}=\frac{{\left(1.96\right)}^{2}.\left(0.657\right).\left(1-0.657\right)}{{\left(0.05\right)}^{2}}. \end{array}$$

SS = 346.28

A total of 692 study participants (346 dengue-positive cases and 346 dengue-negative cases) were recruited as per SS calculation.

### 2.3. Laboratory Analysis

Laboratory diagnosis of dengue was carried by DengueNS1Ag + Ab combo kit (Biotrol Laboratories, India), an ICT-based rapid diagnostic test kit having 96.5% sensitivity and 100% specificity for IgM and 96.7% sensitivity and 100% specificity for IgG (as quoted by the company). Blood sugar, liver function test {LFT: aspartate transaminase (SGOT), alanine transaminase (SGPT), alkaline phosphatase, total protein, albumin, total bilirubin, direct bilirubin} and renal function test (RFT: urea, creatinine, sodium, potassium) were measured by the autoanalyzer (Beckman AU480). Complete blood count (CBC) was analyzed by Automated Coulter Counter (HORIBA XL80) and prothrombin time by Quick PT methods, which is based on the technique described by Quick and his colleagues.

A self-designed structured proforma was used to record the data of the patients including the demographic, clinical symptoms, hematological and biochemical measurements. Sample handling and processing was done following the standard protocol following aseptic technique.

### 2.4. Statistical Analysis

Data was entered in MS Excel 2010 and analyzed with Statistical Package for Social Sciences (SPSS version 22.0). The normality of the data was checked using the Kolmogorov–Smirnov test. Descriptive statistics was used to express categorical variables (clinical features, dengue serology, altered hematology profile, altered LFT profile, altered RFT profile) in frequency and percentage while continuous variables (single laboratory parameters) in median and interquartile range. Inferential statistics, Mann–Whitney U test was used to analyze laboratory parameters between dengue-positive and dengue-negative groups. Kruskal–Wallis H test was used to analyze the laboratory parameters between subgroups (NS1 positive, IgM positive, both NS1, and IgM positive) of dengue patients. Any patient with incomplete/missed data was not included in the study.

### 2.5. Ethical Approval

This study was approved by Institutional Review Committee of Institute of Medicine (Ref. No.: 510(6-11) E^2^ 079/080).

## 3. Results

### 3.1. Clinical Characteristics

Clinical symptomatology in the enrolled dengue patients (*n* = 346) showed predominance of fever (94.5%) with other major features like headache (79.8%), myalgia (74.7%), and nausea (58.5%).

### 3.2. Demographic Characteristics

We found slightly higher number of dengue infected females (*n* = 190, 54.9%) than males (*n* = 156, 45.1%). Furthermore, dengue-positive cases were more concentrated in the age group 20–29 years.

### 3.3. Laboratory Result

#### 3.3.1. Dengue Serology

**Figure 1:**
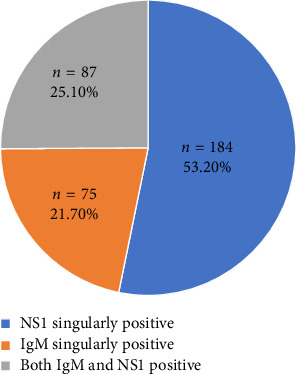
Dengue serology in dengue positive cases.

There were dengue-positive cases with 184 patients having only NS1 positivity, 75 patients having only IgM positivity and 87 patients having both NS1 and IgM positivity. The percentage distribution of dengue serology profile of dengue-positive cases is shown in Figure 1.

**Figure 2:**
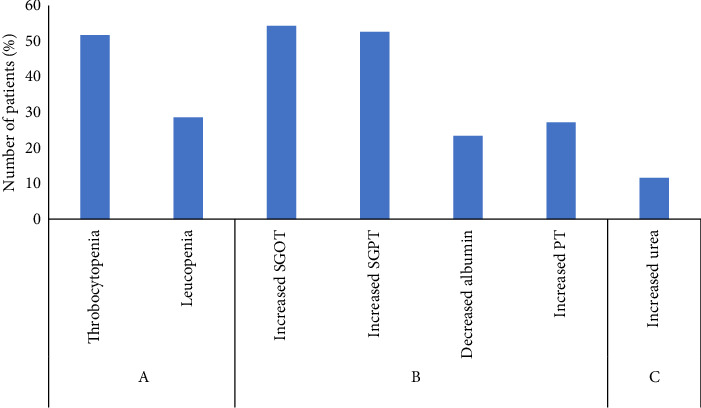
Percentage of dengue patients with A. Altered hematological profile, B. Altered liver function test, and C. Altered renal profile. Altered hematological profile implies thrombocytopenia (count < 150000/cu mm) and leukopenia (count < 4000/cu mm). Altered liver function tests implies SGPT > 35 U/L, SGOT > 35 U/L, serum albumin (< 35 gm/L), PT > 15 s. Altered renal profile implies serum urea > 7.2 mmoles/L.

The incidence of altered laboratory test profile in dengue-positive samples is depicted in Figure 2.

The comparison of hematological and biochemical laboratory findings between the dengue positive and dengue-negative groups are presented in Table 1. Likewise, the comparison of hematological and biochemical laboratory findings within the seropositive subtypes of dengue is presented in Table 2. Leukopenia was more severe in patients with only NS1 positive cases (*p* = 0.008) whereas thrombocytopenia (*p* ≤ 0.001) was more severe in patients with double marker [IgM and NS1] positive cases. Liver function tests showed higher SGPT (*p* = 0.033) and SGOT (*p* = 0.035) in double marker positive group [IgM and NS1] as compared to the other two group [IgM only and NS1 only].

## 4. Discussion

In recent decades, the worldwide prevalence of dengue has increased drastically due to the limitations of existing control strategies like vaccinations and pesticides [21]. Major recurring outbreaks of dengue in Nepal throughout the years 2010, 2013, 2016, 2019, and 2022 indicate a two to 3-year cycle trend. Therefore, it is crucial to have an early diagnosis and appropriate patient care and management. Thus, this study emphasized to record the first baseline data on clinical features, biochemical and hematological parameters of dengue patients. The information obtained is essential for managing dengue patients properly.

In the present study, fever was the most common symptom followed by headache and myalgia. This was in accordance to the study reported by Murmu et al. [22], Mangudkar, Shivnitwar, and Malik [23], and Agrawal et al. [24]. Our study showed that people in the age group of 20–29 years are more susceptible to infection, which is supported by other studies [11, 25, 26]. Among the parameters analyzed, our study demonstrated thrombocytopenia, decreased leukocytes count with neutropenia, lymphocytosis and monocytosis, elevated SGPT and SGOT, increased urea and decreased albumin, increased prothrombin time to be significantly associated with dengue-positive cases.

According to WHO, hematocrit and thrombocytopenia are the two most crucial laboratory values to be assessed during a dengue illness [27]. Numerous researches have demonstrated a strong correlation between dengue severity and thrombocytopenia, which held significance in our setup as well [11, 28–30]. The reduction in platelets might be caused by either reduced platelet production or enhanced platelet destruction through the activation of complement factor C3 and subsequent platelet surface binding of the C5b-9 complex [31]. However, hematocrit did not significantly correlate with IgM or NS1 over the serological course in our study. Due to the fact that our study only included individuals with mild primary active infections, few other studies have shown findings that are similar to our study. Consequently, there is a lower possibility of plasma leakage, which is not indicative of aberrant hematocrit readings [32, 33].

In addition to the decreased platelets, our study showed decreased total leukocyte count with neutropenia, lymphocytosis and monocytosis in dengue-positive patients as compared to dengue-negative patients. In contrast to our study, leukopenia with lymphopenia has been observed in other study [33]. Leukopenia is caused by virus-induced destruction of WBC and on the other hand, suppression of myeloid progenitor cells and leukopenia with monocytosis is explained by monocytes phagocytosing and presenting the antigen to T-helper cells [34, 35].

Antibodies specific to the dengue virus enhance the frequency of virus-infected monocytes in the early stages of dengue infections. As a result, there are more cells expressing dengue virus antigens to lymphocytes. The amount of T-lymphocyte activation is noticeably higher in the middle stages of infection, which is consistent with enhanced antigen presentation, a higher frequency of dengue virus-specific T cells in secondary infection, and faster activation and proliferation of memory T lymphocytes [36].

In our study, leukopenia was more severe in patients with only NS1 positive cases whereas thrombocytopenia was more severe in patients with double marker [IgM and NS1] positive cases. In contrast to these findings, similar study conducted in Nepal reported that both leukopenia and thrombocytopenia were more common in patients with only NS1 positive cases [11].

This study demonstrated the elevated transaminases in dengue patients as compared to dengue-negative patients which were statistically significant. This supports the finding that suggests elevated transaminase levels in dengue-positive cases. Damage to nonhepatic tissues can also raise AST relative to ALT since AST is of both hepatic and nonhepatic origin, whereas ALT is largely of hepatic origin [11, 37, 38]. Additionally, acetaminophen is advised to reduce dengue symptoms; however, even at therapeutic dosages, acetaminophen may temporarily raise transaminase levels [39, 40].

Our study showed decreased level of serum albumin in dengue-positive cases that is 23.4% of total dengue-positive group as compared to dengue-negative group. Similar result was found in a study in India where 26.5% of patients had hypoalbuminemia [41]. Hypoalbuminemia incites worse prognosis in dengue patients, supervised albumin transfusion reverts the progress from dengue shock syndrome [42]. The nonstructural proteins of DENV attaches to heparin sulfate, facilitating leakage of albumin from plasma pools [43]. Complex interactions between the virus and endothelial cells caused by the dengue infection result in endothelial damage, which may increase vascular permeability and compromise the integrity of the barrier. This leakage of plasma proteins explains the low levels of albumin and serum proteins. Low albumin levels thus signal an early entry into the critical period, emphasizing the importance of prompt diagnosis and treatment [44]. We could not prospectively evaluate the outcomes of dengue patients with hypoalbuminemia as compared to normoalbuminemia due to resource constraints.

The serum urea levels were raised in 11.6% of the total dengue-positive cases which is consistent with other study [21]. These could be due to a direct viral effect on the glomerular and tubular cells or as a result of tissue injury caused by deregulated host immune response against the viral antigens [45].

Dengue does not have any specific treatment but early detection and medical cure can prevent fatality associated with its severe form [2]. The impact of dengue on blood, liver, volumic state determines the severity in the patient, and hints on the infecting dengue serotype [46, 47]. The severity of the disease is based on the emergence of a particular serotype of dengue during each dengue outbreak, coinfection with different serotypes or reinfection with a different serotype [48]. Thus, a study like ours provides insights and contributes valuable data on dengue status and its associated complications, would definitely help in the national awareness and surveillance in understanding of dengue with its manifestation and to be vigilant in updating the Nepal's national guidelines and policies of dengue prevention, management, and control that was instigated after 2019 dengue outbreak [49].

### 4.1. Limitations

This study has few limitations; the major being the ICT-based rapid diagnostic method was performed during testing. More sensitive and specific tests like enzyme-linked immunosorbent assay and molecular tests were not performed due to resource constraints. Due to the lack of proper patient's information to categorize dengue fever, we could not classify the patients according to their severity. We would suggest further research in identifying the virus serotypes during the outbreak season to correlate with dengue complications.

## 5. Conclusion

The present study concluded that there was a marked alteration of hematological and biochemical parameters like thrombocytopenia, leukopenia, deranged SGPT, SGOT levels, and high prothrombin time in dengue-positive cases. Leukopenia was more severe in patients with only NS1-positive cases whereas thrombocytopenia was more severe in patients with dual antigen IgM- and NS1-positive cases. Thus, a national awareness to timely approach the hospital and carry out laboratory investigations to be vigilant on the impending complications with dengue infection.

## Figures and Tables

**Table 1:** Comparison of hematological and biochemical parameters between dengue-positive and dengue-negative study population.

Variables	Dengue-positive median [Q1, Q3]	Dengue-negative group median [Q1, Q3]	*p*-value
Age (years)	31 [23, 47]	28 [22, 38.5]	0.011^∗^
Hb (gm%)	13.2 [ 11.5, 14.6]	12.6 [11.5, 14.1]	0.132
PCV %	39.2 [34.9, 43.4]	38.2 [35.7, 41.9]	0.346
RBC (million/cu mm)	4.67 [4.21, 5.17]	4.6 [4.1, 4.8]	0.002^∗^
MCV (fL)	84.8 [80.9, 88.6]	85.0 [82.8, 88.9]	0.017^∗^
MCH (pg)	28.4 [26.7, 29.7]	28.6 [27.4, 30.0]	0.087
MCHC (%)	33.4 [32.3, 34.0]	33.5 [32.6, 34.1]	0.92
Platelets (per cu mm)	147000 [104250, 210250]	197000 [163000, 255000]	**< 0.001**⁣^∗∗^
WBC (per cu mm)	5500 [3900, 7425]	7000 [5300, 9700]	**< 0.001**⁣^∗∗^
Neutrophil (per cu mm)	3744 [2549, 5602]	5402 [3332, 7476]	**< 0.001**⁣^∗∗^
Lymphocyte (per cu mm)	1027 [684, 1536]	1300 [864, 1722]	**0.007**⁣^∗^
Monocyte (per cu mm)	330 [224, 448]	402 [215, 612]	**< 0.001**⁣^∗∗^
Eosinophil (per cu mm)	68[40, 109]	107 [56, 188]	**0.002**⁣^∗^
Sugar (mmol/L)	5.6 [5.0, 6.4]	5.6 [5, 6.3]	0.369
Urea (mmol/L)	3.6 [2.8, 4.9]	3.2 [2.6, 4.1]	**< 0.001**⁣^∗∗^
Creatinine (μmol/L)	71 [59, 90]	69.5 [55, 83]	0.61
Sodium (mEq/L)	136 [134, 137]	136 [133, 137]	0.618
Potassium (mEq/L)	3.8 [3.5, 4]	3.8 [3.6, 4.1]	0.684
Total bilirubin (*μ*mol/L)	12 [9, 17]	13 [8, 16]	0.361
Direct bilirubin (*μ*mol/L)	3 [2, 4]	2 [2, 4]	0.304
SGPT (U/L)	39.5 [21.0, 68.0]	29 [15, 41]	**< 0.001**⁣^∗∗^
SGOT (U/L)	41 [25.0, 68.3]	29 [21, 42]	**< 0.001**⁣^∗∗^
AST/ALT	1.21 [0.87, 1.55]	1.13 [0.91, 1.50]	0.416
ALKP (U/L)	81 [57.7123]	81 [68, 119]	0.152
Total protein (gm/L)	69 [65, 73]	70 [66, 73]	0.44
Albumin (gm/L)	39 [35, 41]	41 [40, 43]	**< 0.001**⁣^∗∗^
PT (seconds)	15 [14, 17]	15 [14, 16]	**< 0.001**⁣^∗∗^
INR	1.25 [1.16, 1.41]	1.25 [1.16, 1.33]	**< 0.001**⁣^∗∗^

*Note:* Bold *p* values are shown as significant.

^∗^Statistically significant at *p* < 0.05.

^∗∗^Statistically significant at *p* < 0.001.

**Table 2:** Comparison of hematological and biochemical parameters between different seropositive dengue patients.

Variables	Only IgM positive (*n* = 75) median [Q1, Q3]	Both IgM and NS1 positive (*n* = 87) median [Q1, Q3]	Only NS1 positive (*n* = 184) median [Q1, Q3]	*p* value
Age (years)	32 [23, 51]	34 [24, 50]	30 [22, 45]	0.198
Hb (gm%)	12.9 [11.5, 14.3]	13.2 [11.6, 15.0]	13.1 [11.3, 14.7]	0.345
PCV %	38.7 [35.4, 42.7]	39.3 [35.2, 44.3]	39.3 [34.0, 43.9]	0.344
RBC (million/cu mm)	4.82 [4.27, 5.18]	4.5 [4.11, 5.29]	4.6 [4.2, 5.17]	0.817
MCV (fL)	84 [80, 88.29]	85.26 [82.0, 89.7]	84.7 [80, 87.5]	0.074
MCH (pg)	28.4 [26.4, 29.3]	28.8 [27.5, 30]	28.2 [26.6, 29.7]	**0.042**⁣^∗^
MCHC (%)	33.3 [32.7, 34.0]	33.6 [32.9, 34.1]	33.3 [32.01,34.0]	0.228
Platelets (per cu mm)	165000 [117000, 234500]	116000 [69000, 16300]	150000 [111250, 215750]	**< 0.001**⁣^∗∗^
WBC (per cu mm)	6500 [4600, 8100]	5300 [4000, 7400]	5100 [3600, 7175]	**0.008**⁣^∗^
Neutrophil (per cu mm)	4428 [3000, 6059]	3567 [2574, 5312]	3516 [2291, 5392]	0.370
Lymphocyte (per cu mm)	1022 [891, 1536]	1092 [748, 1554]	1008 [639, 1535]	0.462
Monocyte (per cu mm)	348 [240, 480]	315 [250, 430]	312 [210, 429]	0.819
Eosinophil (per cu mm)	78 [54, 146]	70 [45, 96]	62 [37, 102]	0.648
Sugar (mmol/L)	5.3 [4.9, 6.3]	5.70 [5.10, 7.0]	5.7 [5.0,6.2]	0.121
Urea (mmol/L)	4.0 [3.0, 5.0]	3.8 [2.9, 6.1]	3.6 [3.0, 4.7]	0.324
Creatinine (μmol/L)	71 [61,83]	70 [57.0, 97.0]	72.5 [58.25, 90.0]	0.993
Sodium (mEq/L)	136 [133, 137]	135 [133, 137]	136 [134, 137]	0.076
Potassium (mEq/L)	3.9 [3.7, 4.1]	3.8 [3.58, 4.0]	3.8 [3.5, 4]	0.134
Total bilirubin (μmol/L)	12 [8, 17]	15.0 [10, 20]	12 [9, 16]	**0.018**⁣^∗^
Direct bilirubin (/L)	2 [2, 4]	3 [2, 5]	3 [2, 4]	**0.010**⁣^∗^
SGPT (U/L)	41 [20, 66]	56 [26, 77]	34 [19, 63.75]	**0.033**⁣^∗^
SGOT (U/L)	35 [26, 61]	53 [27, 86]	38.5 [24, 66.75]	**0.034**⁣^∗^
AST/ALT	1.15 [0.84, 1.40]	1.20 [0.85, 1.58]	1.21 [0.914, 1.58]	0.246
ALKP (U/L)	106 [69, 128]	77 [55, 119]	79 [55, 117]	**0.036**⁣^∗^
Total protein (gm/L)	69 [67, 73]	68 [65, 71]	69 [65, 74]	0.171
Albumin (gm/L)	39 [35.5, 41]	39 [35, 42]	38.5 [35, 41.75]	0.910
PT (seconds)	15 [15, 16]	15 [14, 17]	15 [15, 17]	0.589
INR	1.25 [1.25, 1.33]	1.33 [1.16, 1.41]	1.25 [1.16, 1.41]	0.706

*Note:* Bold *p* values are shown as significant.

^∗^Statistically significant at *p* < 0.05.

^∗∗^Statistically significant at *p* < 0.001.

## Data Availability

The data that support the findings of this study are available from the corresponding author upon reasonable request.

## Nomenclature

Hb: Hemoglobin
RBC: Red blood cell
HCT: Hematocrit
MCV: Mean cell volume
MCH: Mean cell hemoglobin
MCHC: Mean cell hemoglobin concentration
TLC: Total leukocyte count
ALP: Alkaline phosphatase
SGPT: Serum glutamic pyruvic transaminase
SGOT: Serum glutamic-oxaloacetic transaminase
ORC: Crude odds ratio
ORA: Adjusted odds ratio
PCR: Polymerase chain reaction
